# Unraveling the Fecal Microbiota and Metagenomic Functional Capacity Associated with Feed Efficiency in Pigs

**DOI:** 10.3389/fmicb.2017.01555

**Published:** 2017-08-15

**Authors:** Hui Yang, Xiaochang Huang, Shaoming Fang, Maozhang He, Yuanzhang Zhao, Zhenfang Wu, Ming Yang, Zhiyan Zhang, Congying Chen, Lusheng Huang

**Affiliations:** ^1^State Key Laboratory of Pig Genetic Improvement and Production Technology, Jiangxi Agricultural University Nanchang, China; ^2^College of Bioscience and Engineering, Jiangxi Agricultural University Nanchang, China; ^3^National Engineering Research Center for Breeding Swine Industry, Guangdong Wens Foodstuff Co. Ltd. Xinxing, China

**Keywords:** feed efficiency, gut microbiota, metagenome, 16S rRNA gene, swine

## Abstract

Gut microbiota plays fundamental roles in energy harvest, nutrient digestion, and intestinal health, especially in processing indigestible components of polysaccharides in diet. Unraveling the microbial taxa and functional capacity of gut microbiome associated with feed efficiency can provide important knowledge to improve pig feed efficiency in swine industry. In the current research, we studied the association of fecal microbiota with feed efficiency in 280 commercial Duroc pigs. All experimental pigs could be clustered into two enterotype-like groups. Different enterotypes showed the tendency of association with the feed efficiency (*P* = 0.07). We further identified 31 operational taxonomic units (OTUs) showing the potential associations with porcine feed efficiency. These OTUs were mainly annotated to the bacteria related to the metabolisms of dietary polysaccharides. Although we did not identify the RFI-associated bacterial species at FDR < 0.05 level, metagenomic sequencing analysis did find the distinct function capacities of gut microbiome between the high and low RFI pigs (FDR < 0.05). The KEGG orthologies related to nitrogen metabolism, amino acid metabolism, and transport system, and eight KEGG pathways including glycine, serine, and threonine metabolism were positively associated with porcine feed efficiency. We inferred that gut microbiota might improve porcine feed efficiency through promoting intestinal health by the SCFAs produced by fermenting dietary polysaccharides and improving the utilization of dietary protein. The present results provided important basic knowledge for improving porcine feed efficiency through modulating gut microbiome.

## Introduction

Feed costs account for 60–70% of the total costs involved in pig production. Improving feed efficiency has been an important task of commercial pig breeding. Many factors affect porcine feed efficiency, including genetics, diet, diseases, and management (rearing environment; Woltmann et al., 1992; Armstrong et al., 2000; Saintilan et al., 2015). The studies on porcine gut microbiome suggested that gut microbiota should also have a profound effect on porcine feed efficiency (Lamendella et al., 2011; Vigors et al., 2016).

Animal gastrointestinal microbiota is a heterogeneous ecosystem and dominated by bacteria. Several previous studies reported the structure and functional capacity of porcine gut microbiome. Lamendella et al. found that *Prevotella* spp. and the function term of carbohydrate metabolism dominated the swine fecal metagenome (Lamendella et al., 2011). Kim et al. showed microbial population shifts representing both microbial succession and changes in response to the use of tylosin (Kim et al., 2012). Looft et al. identified numerous microbial genes degrading plant cell wall components in porcine large intestine (Looft et al., 2014). McCormack et al. identified several gut microbes potentially associated with porcine feed efficiency (McCormack et al., 2017). Bacterial communities in piglets could be separated into two different enterotype-like clusters, primarily distinguished by unclassified *Ruminococcaceae* and *Prevotella* levels (Mach et al., 2015). Ramayo-Caldas et al. (2016) defined the interaction network of gut microbiota in pigs, and found that the enterotype-like clustering was significantly associated with porcine growth traits.

Feed efficiency is generally expressed as feed conversion ratio (FCR) or residual feed intake (RFI, also called net feed efficiency). RFI is defined as feed intake adjusted for the maintenance requirements and body weight gain. It specifically captures the efficiency of feed utilization independent from production needs (Gilbert et al., 2017). A variety of factors influence porcine RFI, such as digestion, metabolism, and host body conditions (health). Nutrient digestibility is an important factor influencing porcine RFI. A previous study found a higher total tract digestibility of dry matter, nitrogen, and gross energy in the low RFI pigs (Harris et al., 2012). The microbes in the gastrointestinal tract provide numerous biological activities that the host lacks (Turnbaugh et al., 2006). Gut microbiota plays fundamental roles in nutrient uptake, energy harvest, and carbohydrate metabolism (Greenblum et al., 2012), especially in processing indigestible components of most mammalian diet such as plant polysaccharides (Backhed et al., 2005). Metabolic enzymes encoded by microbial genomes degrade dietary polysaccharides and cellulose that cannot be digested by host into short chain fatty acids (SCFAs; Turnbaugh et al., 2006). The microbiome in the distal end of intestinal tract contains gene clusters related to the synthesis of essential amino acids and vitamins (Delzenne and Cani, 2011). Intestinal dysbacteriosis leads to inflammation, reduction in absorptive capacity and low efficiency of feed utilization. However, to the best of our knowledge, there are only few studies about the association between gut microbiome and feed efficiency in pigs. Understanding how the intestinal microbial community alters the host's capacity for metabolism and energy harvest is a significant issue for improving feed efficiency in the pig production industry.

In this study, we used 16S rRNA gene sequencing and shotgun metagenomic sequencing to uncover the effects of host and environmental factors on the diversity of porcine gut microbial communities, and to identify gut microbiota, and the potential function capacity correlated with swine feed efficiency.

## Materials and methods

### Animal feeding and management

A total of 280 commercial Duroc pigs including 169 males and 111 females from Shahu farm were used in this study. The experimental pig cohort contained 24 pairs of half-siblings and 75 pairs of full-siblings. All animal works were conducted according to the guidelines for the care and use of experimental animals established by the Ministry of Agriculture of China. The project was specially approved by Animal Care and Use Committee (ACUC) in Jiangxi Agricultural University. All experimental pigs were weaned at the age of 28 days and raised under the similar feeding and management manners before the fattening stage, such as man-controlled farm conditions and the same commercial formula diet. When their body weights reached 30 kg (at the age of 70–90 days; Supplementary Table 1), all pigs were transferred to the fattening house which was comprised of 36 pens. Each pen housed 8–12 pigs. During the fattening stage, all experimental pigs were provided the same commercial formula diet which mainly contained corn, soybean meal, soybean oil, and calcium hydrophosphate, and consisted of 2,440 kJ digestible energy, 17% crude protein, 0.94% lysine, 0.9% calcium, 0.34% phosphorus, and 0.16% salt. Diet was available *ad libitum* from automatic feeding trough (Osborne Industries, USA), which separately recorded daily feed intake and daily body weight gain of each pig. All experimental pigs were healthy and not received any antibiotic treatment during the period of measurement.

### Phenotypic measurement and fecal sample collection

The following model was applied to calculate RFI: *DFI* − *sex* − *batch* − *pen* = α + β_1_*MBW* + β_2_*LMC* + *e*, where *DFI* refers to average daily feed intake; α is intercept; *MBW* represents metabolic body weight; *LMC* is lean meat content; and *e* represents the uncontrolled error. β_1_ and β_2_ indicate the corresponding estimated effects for *MBW* and *LMC*. For calculating the *MBW*, we used the formula: $\text{MBW}=\frac{\left(w_{1}^{1.75}-w_{0}^{1.75}\right)}{1.75\times \left(w_{1}-w_{0}\right)}$ (*W*_0_ = body weight at start; *W*_1_ = body weight at end) as described previously (Haer et al., 1993). The RFI-values obtained from the age of day 100 to day 160 (intermediate stage of phenotypic measurement) were used for further association analysis between the RFI and the relative abundance of bacteria. The fecal samples were collected from each animal's anus at the age of day 140. After dipped in liquid nitrogen, all samples were transferred into −80°C freezer until use. We chose 18 fecal samples for metagenomic sequencing, including nine samples from the pigs with the low RFI-values (high feed efficiency; 5 ♂ and 4 ♀; RFI: −0.22 ± 0.08) and nine samples with the high RFI-values (low feed efficiency; 4 ♂ and 5 ♀; RFI: 0.094 ± 0.068; Supplementary Figure 1). The 18 animals included five pairs of full-siblings having opposite RFI phenotypes.

### High-throughput sequencing of bacterial 16S rRNA gene

Fecal DNA was extracted using QIAamp Fast DNA Stool Mini Kit (Qiagen, Germany) according to the manufacturer's instructions. DNA samples were diluted to 1:10, and then 1-μl diluted DNA sample was used for PCR. The conserved primers 515F (5′-GTGCCAGCMGCCGCGGTAA) and 806R (5′-GGACTACHVGGGTWTCTAAT) were used to amplify the V4 hypervariable region of 16S rRNA gene. PCRs were performed in 30 cycles at 60°C of annealing temperature, and the products were separated by gel electrophoresis. After purification, the PCR products were used to construct the libraries, and then sequenced on a MiSeq platform (Illumina, USA) at the Beijing Genomics Institute (BGI, China).

### Taxonomic classification and diversity analysis

To obtain the clean sequence reads, the primer and barcode sequences, and the low quality reads were filtered out from raw data (Fadrosh et al., 2014). Paired-end clean sequence reads were then assembled into tags with the overlapping relationship by QIIME (v1.80) (Caporaso et al., 2010). To avoid the effect of the sequencing depth on the measurement of microbial composition (Hughes and Hellmann, 2005), we rarefied the library size to 10,000 tag-depth per sample using the rarefy function in R package (Version 3.2.3). Tags were clustered into operational taxonomic units (OTUs) at the 97% similarity using USEARCH software (v7.0.1090) (Majaneva et al., 2015). Taxonomic assignments for the 16S rRNA gene sequences were made using the RDP classifer program (v2.2) (Wang et al., 2007). MOTHUR (v1.31.2) (Venkataraman et al., 2015) was used to calculate alpha-diversity (Gihring et al., 2012). Canonical Correspondence Analysis (CCA) was used to examine possible correlations between the relative abundance of bacteria and the variables of environmental and host factors, including pen, kinship and sex using the vegan package in R (Dixon, 2004). Unweighted UniFrac distances were calculated to compare beta-diversity of microbial community between different statuses of gender, kinship, and pen by QIIME (Lozupone and Knight, 2005).

### Individual enterotype clustering and association with host feed efficiency

Enterotype analysis of the experimental pig cohort was done using the methodology described by Arumugam et al. (2011). Briefly, samples were clustered using *Jensen-Shannon divergence (JSD)* distance and the *Partitioning Around Medoids (PAM)* clustering algorithm based on the relative abundances of bacteria at the genus level. To determine the optional number of clusters, the *Calinski-harabasz* (*CH*) *Index* was evaluated from 2 to 20 clusters. The cluster number that had the highest CH index and was also validated by the silhouette coefficient was chosen as the optimal number. To test a possible effect of predicted enterotype-like clusters on host feed efficiency (RFI), the comparison analysis of the RFI-values between enterotypes was performed using Wilcox test.

### Association study using two-part model

We first filtered out those OTUs which had <0.01% of relative abundance and were presented in <5% of the tested samples from further association analysis. Because most bacteria were not presented in many samples and the distribution of the relative abundances of OTUs did not match a normal distribution, we adopted a two-part model to analyze the association between the relative abundances of bacteria and the RFI-values (Fu et al., 2015). This model accounts for both binary (detected/undetected) and quantitative features of gut microbial abundances and overcomes the problem of a non-normal distribution. The binary analysis was to test for association of detecting a microbe (detected/undetected) with RFI. The binary feature (*b*) of a microbe was coded as 1 for detected or 0 for undetected for each sample. The binary model is described as: *y* = β_1_ *b* + *e*, where *y* refers to the RFI-value after adjusting for sex, batch, and pen, *b* represents a binary feature, β_1_ is the estimated effect for existence or inexistence of bacteria, and *e* is the residuals. The quantitative analysis evaluated the association between the RFI-values and the relative abundances of bacteria in the tested samples. The quantitative model is set as: *y* = β_2_ *q* + *e*, where *y* refers to the RFI-value per sample after adjusting for sex, batch, and pen, *q* represents the abundance of a microbe, β_2_ is the estimated effect of bacterial abundance, and *e* is the residuals. To further evaluate the effect of both binary and quantitative analysis, a meta *P*-value was calculated using an unweighted Z method. The minimum of *P*-values from the binary analysis, quantitative analysis, and meta-analysis was set as the final association *P*-value per microbe and RFI pair. The association Z scores were determined based on the Z distribution. A negative or positive Z score represents the negative or positive association direction, respectively. To correct the false positive, we performed a multiple hypothesis testing to control the false discovery rate with R packages. The statistical cutoff of false discovery rate (FDR) < 0.05 was set as the significance threshold.

### Metagenomic sequencing analysis

Metagenomic sequencing of the 18 fecal DNA samples were performed using Hiseq 2,500 platform (Illumina, USA). Libraries for metagenomic sequencing were constructed following the manufacturer's instruction (Illumina, USA). We used the same protocol as described by Qin et al. to perform cluster generation and sequencing (Qin et al., 2012). The bioinformatics analyses of metagenomic sequencing data were performed following the standard protocol established by Qin et al. (2012). In brief, those low-quality sequence reads with more than three unknown bases, continuous low-quality bases, or the adapter and duplication pollution were excluded from further analyses. *De novo* assembly of high quality short reads was performed by SOAP *denovo* assembler (v2.21) (Luo et al., 2012). We used USEARCH software (v7.0.1001) to exclude the redundant contigs. The non-redundant contigs were blasted against the nucleotide sequences of bacteria, fungi, viruses, and archaebacteria in the NCBI nucleotide database by LCA algorithm (Huson et al., 2011). We further checked the annotated bacteria carefully, and excluded those bacteria that don't obviously belong to the host-associated organisms from further analysis.

MetaGeneMark (v2.10) was applied to predict a whole range of ORFs from the contigs with more than 300 bp in length (Zhu et al., 2010). Cd-hit software (version 4.6.1) was used to exclude the redundant genes from all predicted ORFs (Li and Godzik, 2006). The non-redundant gene set was then aligned to the protein sequences of KEGG (Kyoto Encyclopedia of Genes and Genomes) pathways (Kanehisa et al., 2004, 2006). Metastats software was used to compare the relative abundances of microbial species between high and low RFI pigs (Paulson et al., 2011). The comparison of KEGG pathways between high and low RFI pigs were performed by STAMP software. To identify the KEGG genes that were associated with porcine RFI, univariate general linear model was used to find the KEGG genes with a *P* < 0.05. And then, the spare partial least squares discriminant analysis (sPLS-DA) was used to identify the key KEGG genes related to the RFI (Cao et al., 2012). In the sPLS-DA analysis, three components and 10 KEGG genes for each component were selected as the optimal parameters.

## Results

### Phenotypic values of porcine RFI in the experimental cohort

All experimental pigs were separately recorded daily feed intake and daily body weight gain from 30 kg (~70–90 days of age) to 100 kg of body weight (~170–190 days of age), which was the fastest growth stage of pigs. To avoid the effect of environmental adaptation of experimental pigs with automatic feeding trough on the phenotypic values, we used the RFI-values calculated from day 100 to 160 (intermediate stage of phenotypic measurement). The distributions of daily feed intake, daily weight gain and RFI-values in the experimental cohort were shown in Supplementary Table 1. The average RFI-value for the experimental pig cohort was −0.026 ± 0.17 (mean ± SD).

### Taxonomic distribution of fecal microbiota in the experimental pigs

All 280 experimental pigs were performed the 16S rRNA gene sequencing. The sequencing data were submitted to the SRA database in NCBI with the accession number SRR5062190. After quality control, we obtained an average of 31,248 high quality tags for each sample. Based on the 97% sequence similarity, we obtained an average of 759 OTUs for each individual. As shown previously in pigs (Isaacson and Kim, 2012; Looft et al., 2014), the phylogenetic composition of fecal microbial community was dominated by *Bacteroidetes, Firmicutes, Proteobacteria*, and *Spirochaetes* at the phylum level (Supplementary Figure 2). At the genus level, we identified a total of 74 bacterial genera. *Prevotella, Lactobacillus*, and *Treponema* were the three most abundant genera (Supplementary Figure 2).

To investigate the microbial composition at the species level and the function capacity of gut microbiome, we performed shotgun metagenomic sequencing in 18 fecal samples from nine boars and nine gilts (methods). The sequence assembly analysis of 18 samples produced a total of 4.73 million contigs with an average length of 875 bp and an average N50 length of 1,135 bp (Supplementary Table 2). The phylogenetic composition of fecal microbiota was determined by blasting the Scaftigs to NCBI nucleotide (NT) database. Similar to the result obtained in the 16S rRNA gene sequencing analysis, *Bacteroidetes, Firmicutes*, and *Proteobacteria* were the three most abundant phyla. At the species level, a total of 2,039 bacterial species were detected in all 18 samples. *Prevotella ruminicola* was the most abundant bacterium in the tested samples. It was strange that we found some bacterial species belonging to the free-living soil-dwelling bacteria. We judged that this should be caused by the eating behaviors of pigs. However, these bacteria had very low relative abundances (<0.02% in total; Supplementary Table 3). We excluded these bacteria from further analysis.

**Figure 1:**
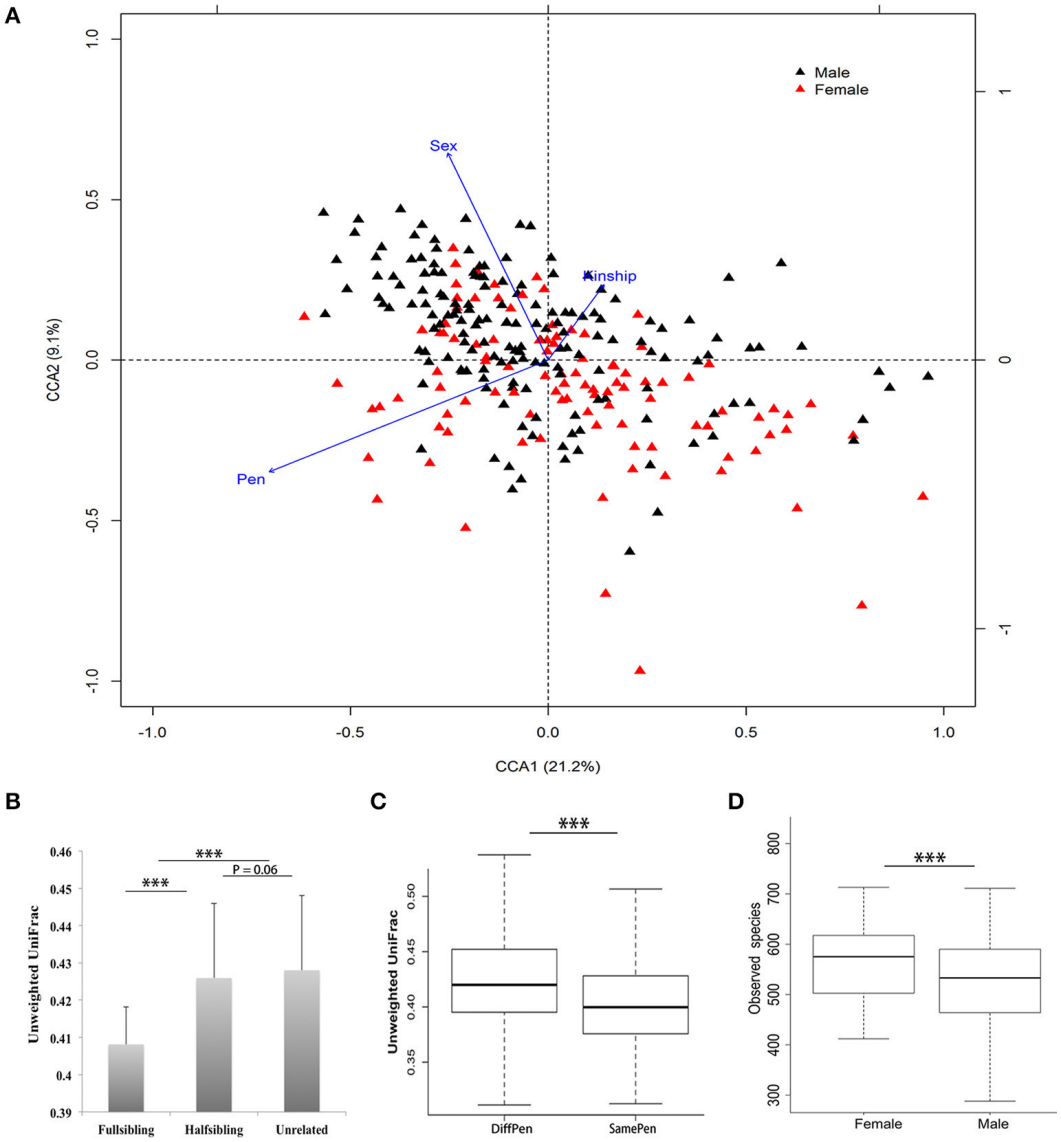
Environmental and host factors affecting porcine fecal microbial community structure. **(A)** Canonical Correspondence Analysis (CCA) found significant effects of pen, sex and kinship on fecal microbial community structure. **(B)** Comparison of Unweighted UniFrac distance among full-siblings, half-siblings, and unrelated individuals. **(C)** Pen shows significant effect on fecal microbial community. The pigs housed in the same pen showed a higher similarity of microbial composition than pigs in different pens. **(D)** Significant difference of alpha-diversity of fecal microbial community between boars and gilts (^***^P < 0.001 for Student's *t*-test; mean ± SEM).

We observed a substantial animal-to-animal variation in microbial composition. For examples, the average abundance of the *Bacteroidetes* was 64.21%, ranging from 26.81% to 86.67%; The *Firmicutes* was 28.22%, which varied from 11.23% to 65.84% (Supplementary Figure 2). A CCA analysis in the tested samples found that host and environmental factors including pen, kinship, and sex had significant effects on porcine fecal microbial composition (Figure 1). Beta-diversity analysis revealed that full-siblings had a higher similarity of bacterial community structure than half-siblings and unrelated individuals. Compared to the pigs in the different pens, the pigs reared in the same pen showed the significantly higher similarity of microbial communities in Unweighted UniFrac metric analysis (*P* < 0.01). Furthermore, gilts had a higher number of observed species than boars (Figure 1).

### Enterotype and association with porcine feed efficiency

**Figure 2:**
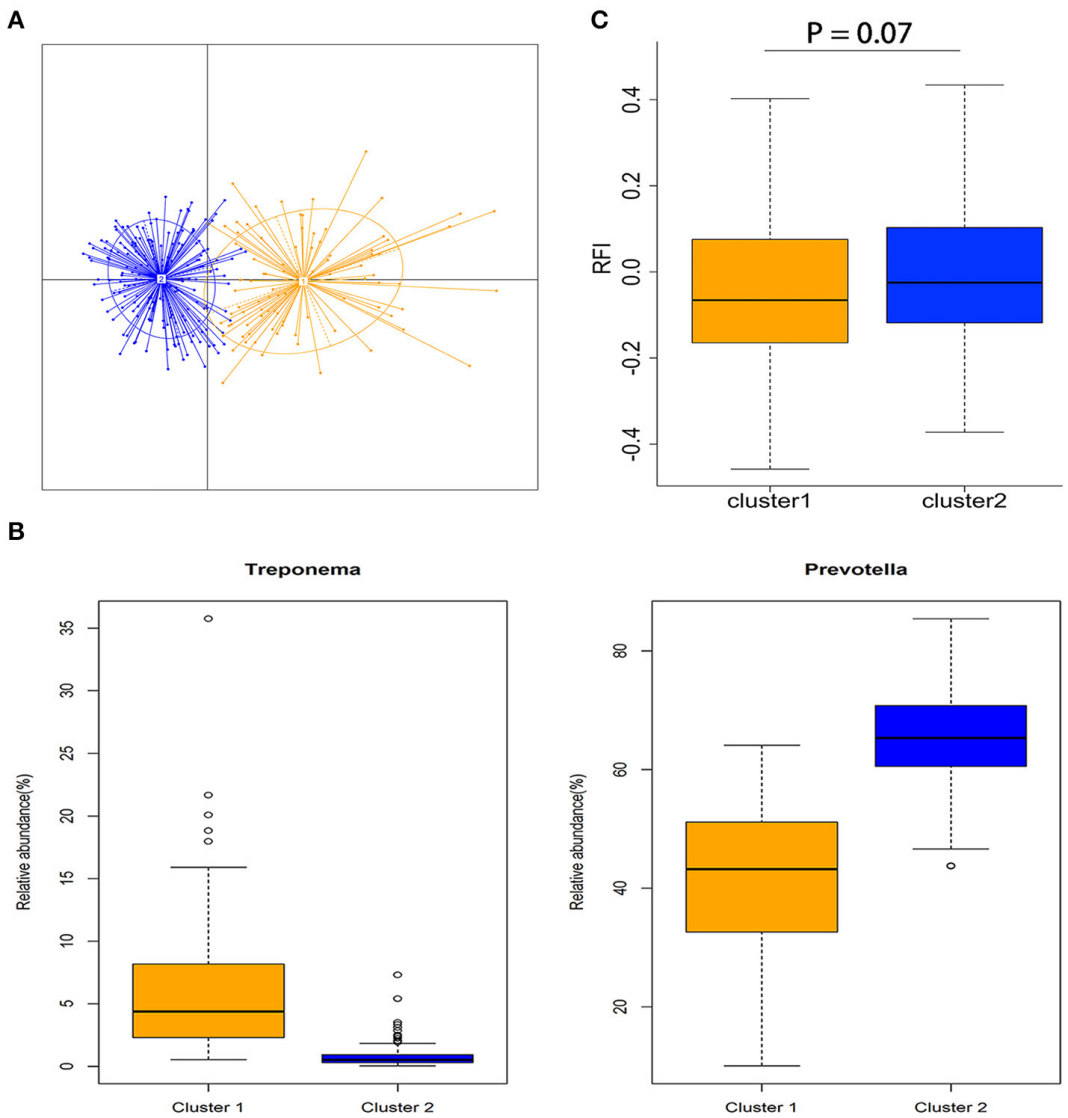
Enterotype-like clustering in a cohort of 280 experimental pigs and association with porcine feed efficiency. **(A)** Enterotype-like clustering of 280 pigs. **(B)** Relative abundances of the main contributors of each enterotype. Orange color represents the enterotype cluster 1 and blue color indicates the enterotype cluster 2. **(C)** Association of enterotype-like clusters with porcine feed efficiency (Cluster 1: *n* = 90 and cluster 2: *n* = 190).

All 280 samples were clustered into two enterotype-like groups (Figure 2A). The enterotypes 1 and 2 were dominated by *Treponema* and *Prevotella*, respectively (Figure 2B). We evaluated the relationship between enterotype and porcine feed efficiency, and observed the tendency of association between the enterotype and porcine RFI (*P* = 0.07; Figure 2C). The pigs clustered into enterotype 1 tended to have the lower RFI-values than those clustered into enterotype 2.

### Microbial taxa associated with porcine feed efficiency

We used a two-part model to perform the association analysis between gut bacteria and porcine RFI. We didn't detect any OTUs significantly associated with the RFI at FDR < 0.05 in the experimental population. But at the threshold of *P* < 0.01 and FDR < 0.1, we identified 31 OTUs showing the tendency of associations with porcine RFI (Supplementary Table 4). Most of the RFI-associated OTUs were annotated to the orders *Clostridiales* and *Bacteroidales*, including 7 OTUs to the *Ruminococcaceae*, 5 OTUs to the *Prevotella*, 4 OTUs to the *Faecalibacterium*, 3 OTUs to the *Christensenellaceae*, 2 OTUs to the *Clostridiales*, 2 OTUs to the *Bacteroidales*, and 2 OTUs to the *Lachnospiraceae*. The most significant association was identified at the OTU277 (*Christensenellaceae, P* = 4.18 × 10^−5^). The OTUs annotated to *Ruminococcaceae, Christensenellaceae, Lachnospiraceae*, and *Akkermansia* had positive associations with porcine feed efficiency (low RFI), while the OTUs annotated to *Prevotella*, and *Faecalibacterium* had negative relationships to feed efficiency.

**Figure 3:**
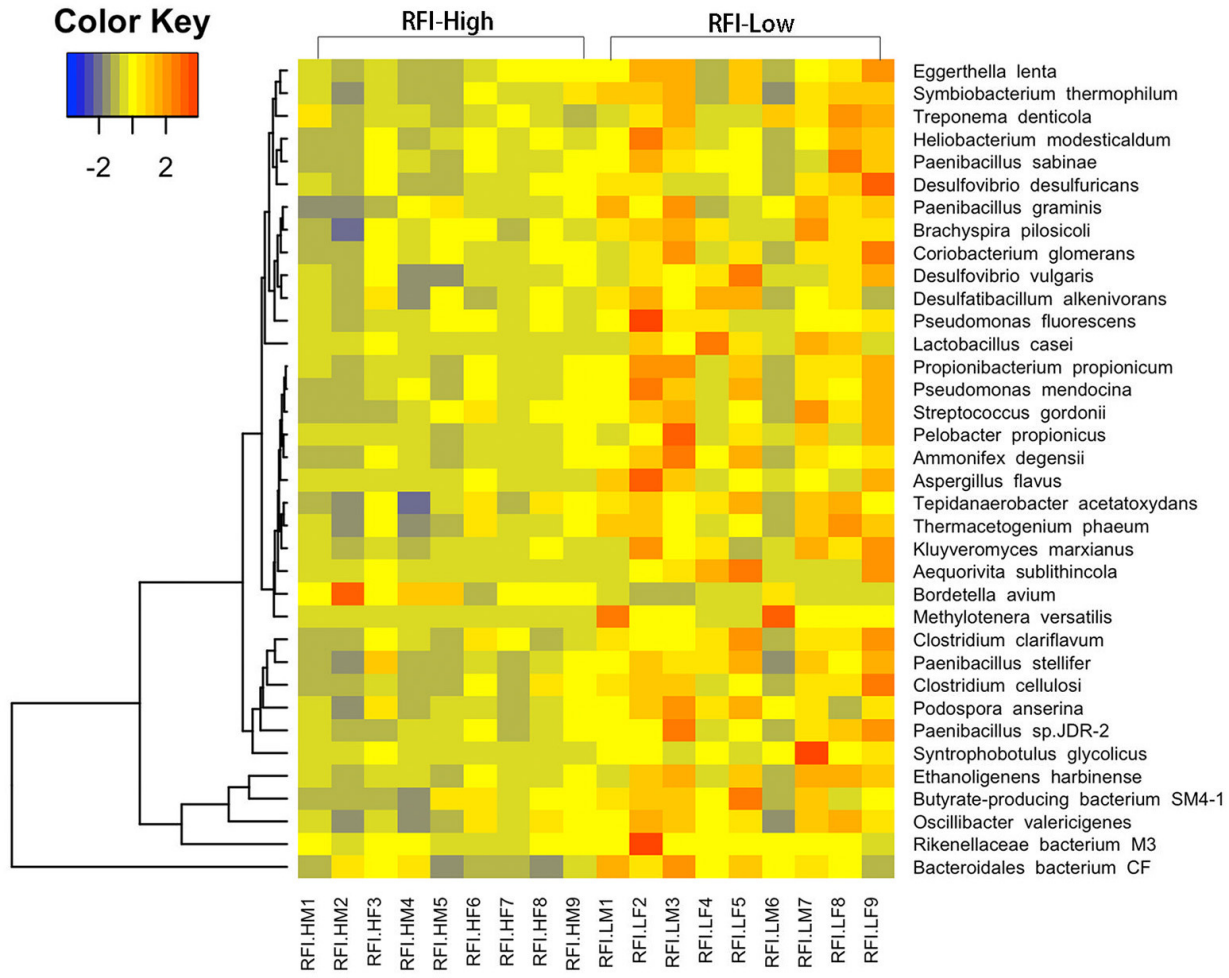
Heatmap of bacterial species showing different enrichment between high and low RFI pigs in the metagenomic sequencing analysis. The X-axis shows the sample IDs, e.g., RFI.HM9 representing the sample 9, a male with high RFI-value; RFI.HF8: the sample 8, a female with high RFI-value; RFI.LF9: the sample 9, a female with low RFI-value, and so on. The Y axis represents the microbial species.

We were further dedicated to identify the RFI-associated bacteria at the finer level with metagenomic sequencing data. We did not identify any RFI-associated bacterial species at FDR < 0.05 level, but a total of 36 bacterial species showed potential associations with porcine RFI at *P* < 0.05 (FDR < 0.2; Figure 3, Supplementary Table 5). We identified four bacterial species from the *Clostridiales* including *Ethanoligenens harbinense, Syntrophobotulus glycolicus, Clostridium clariflavum*, and *Clostridium cellulosi*, and two species from the *Bacteroidales* including *Rikenellaceae bacterium M3*, and *Bacteroidales bacterium CF* showing the enrichments in pigs with high feed efficiency (low RFI-values). Additionally, *Pseudomonas fluorescens, Pseudomonas mendocina, Treponema denticola, Desulfovibrio vulgaris, Desulfovibrio desulfuricans*, and *Lactobacillus casei* were also enriched in pigs with high feed efficiency. We only identified *Bordetella avium* that was overrepresented in pigs with low feed efficiency (Figure 3).

### Functional capacity profiling of gut microbiome related to feed efficiency based on metagenomic sequencing

**Figure 4:**
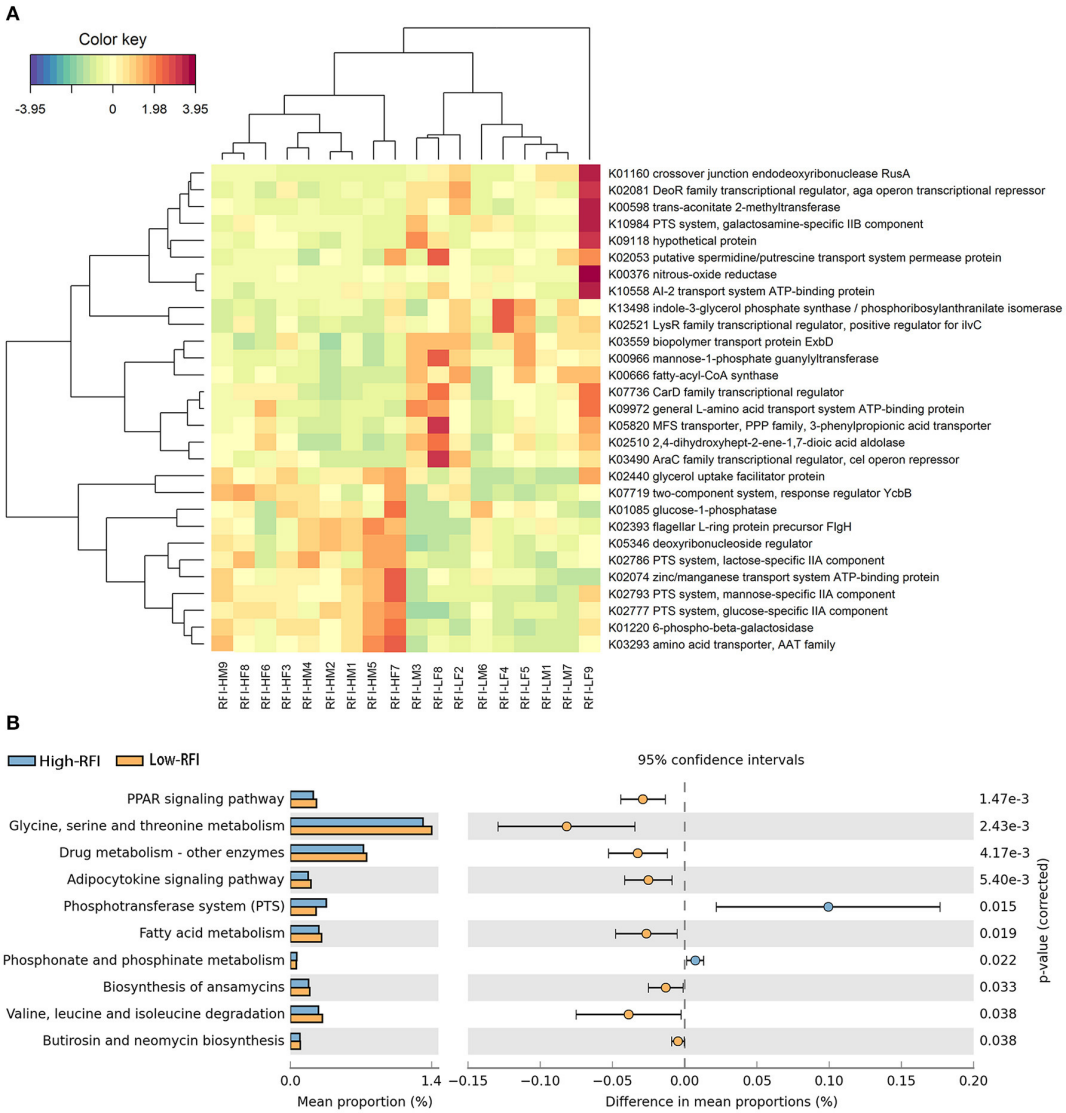
The KEGG function terms showing different enrichment between high and low RFI pigs. **(A)** Heatmap of KEGG orthologs showing different enrichments between high and low RFI pigs. The X-axis shows the sample IDs, e.g., RFI.HM9 representing the sample 9, a male with high RFI-value; RFI.HF8: the sample 8, a female with high RFI-value; RFI.LF9: the sample 9, a female with low RFI-value, and so on. The Y-axis represents the KEGG orthologies. **(B)** Differential KEGG pathways between high and low RFI pigs detected by STAMP software.

Functional capacity of gut microbiome related to porcine RFI was investigated with metagenomic sequencing data. Functional capacity was inferred from the annotation of ORFs predicted from the assembled contigs. A total of 3,452,610 ORFs were found with an average length of 514 bp. We classified these predicted genes by aligning them to the KEGG database. A total of 4,327 KEGG genes were identified and assigned to 329 KEGG pathways. We first analyzed the differential KEGG genes between high and low RFI pigs, and identified 29 KEGG Orthologies (KOs) showing different enrichments (Figure 4A, FDR < 0.05). Eleven out of these 29 KOs were enriched in the pigs with low feed efficiency (high RFI-values), including those KOs associated with metabolism and transport of monosaccharide (K01220, K02777, K02793, K02786, and K01085), and signal transduction (K07719). The other 18 orthologies had the higher abundances in those pigs with high feed efficiency, such as nitrogen metabolism (K00376), amino acid metabolism (K13498 and K02510) and transport system (K02053, K10558, K09972, and K05820; Figure 4A).

We further investigated the KEGG pathways associated with porcine RFI. We identified a total of 10 KEGG function terms showing distinct enrichments between low and high RFI pigs (Figure 4B). Only bacterial phosphotransferase system (PTS), and phosphonate and phosphinate metabolism were more abundant in the pigs with high RFI-value, while the other eight pathways, such as PPAR signaling pathway, amino acid metabolism, fatty acid metabolism, and adipocytokine signaling pathway were enriched in those individuals with low RFI-value.

## Discussion

In this study, we investigated the fecal microbial composition of commercial Duroc pigs, and systematically evaluated the association of gut microbiome with pig feed efficiency from both enterotype and microbial taxa with 16S rRNA gene sequencing and shotgun metagenomic sequencing data. To the best of our knowledge, this is the first study to evaluate the association of gut microbiota with porcine feed efficiency based on both 16S rRNA gene and metagenomic sequencing data in a large-scale of sample size.

Consistent with the previous reports (Lamendella et al., 2011; Looft et al., 2014), *Firmicutes* and *Bacteroidetes* were the most abundant phyla in fecal microbiota of pigs. But significant difference existed in the relative abundance of each bacterial phylum among 280 samples, suggesting the animal-to-animal variation of phylogenetic composition. We found significant effects of pen, sex, and kinship on gut microbial composition (Figure 1). The pen effect should be caused by predictable differences in the colonization process between pigs and subtle differences in their environments (Dethlefsen et al., 2007). The same result was also observed in mice, where separating littermates into different cages can drive the differences in their microbiota (Ley et al., 2005). Study using human twins (Goodrich et al., 2014), and the comparisons between mouse lines (Campbell et al., 2012) and between pig breeds (Pajarillo et al., 2014) have yielded the informative results about heritability of gut microbiota. This can be used to explain the effect of kinship on pig fecal microbial composition observed in this study. Host gender also showed the significant effect on porcine gut microbiota. Previous study reported that puberty affected the microbiotal composition of males, which became less diverse than the female microbiota. This distinct diversity may be caused by host reproduction hormones, such as estrogen and androgen, which regulate the microbial composition and the activity of gut microbiota (Kanehisa et al., 2006; Li and Godzik, 2006; Goodrich et al., 2014). Metagenomic sequencing analysis identified some bacterial species that are not the host-associated organisms. All of these species belong to the free-living soil-dwelling bacteria. However, all samples were collected from the pig anus directly. The possible reason for this contamination should be the feeding habits of pigs.

Two previous studies suggested that porcine gut bacterial communities could be divided into enterotype-like clusters. Different enterotypes were significantly associated with porcine growth traits (Mach et al., 2015; Ramayo-Caldas et al., 2016). In this study, the 280 experimental pigs could be assigned into two enterotype-like clusters. Indeed, we observed the tendency of association between porcine feed efficiency and the enterotype (*P* = 0.07). However, there have been the controversies about enterotype. Knights et al. suggested that enterotype does not regard the individual's position along the spectrum of intra-cluster variation, and a predictive model using taxon-relative abundances would be more effective than a model using cluster labels from unsupervised enterotype clustering (Knights et al., 2014).

Although we did not identify any RFI-associated OTUs at FDR < 0.05 level, some OTUs showed the tendency associated with porcine RFI. Most of these OTUs were annotated to the *Clostridiales*, belonged to the butyric acid-producing bacteria and showed positive associations with feed efficiency. For examples, *Ruminococcaceae* is capable of producing short-chain fatty acids (SCFA) *via* fermenting dietary polysaccharide (Flint et al., 2008). *Christensenellaceae* produces volatile fatty acids as the end products of fermentation (Morotomi et al., 2012). Metagenomic sequencing analysis also identified several RFI-associated bacterial species belonging to the bacterial families that were detected to associate the RFI in the 16S rRNA gene sequencing data, e.g., two cellulolytic species including *C. clariflavum* and *C. cellulosi*, and two bacterial species from the genus *Desulfovibrio* showed the tendency associated with the high feed efficiency. The cellulolytic species could degrade the dietary indigestible cellulose into SCFAs, and the *Desulfovibrio* has the ability of consuming molecular hydrogen produced by fermentation to keep the fermentation reaction going (Gibson et al., 1993). The major ingredients of formula diet provided to the experimental pigs in this study included fiber-enriched corn and high-protein soybean. Therefore, we hypothesized that gut microbiome of the high feed-efficiency pigs might have a greater ability to utilize the dietary indigestible cellulose. The SCFAs produced by fermenting dietary polysaccharide are the preferred energy source rather than glucose and lactose for colonic mucosa (Pryde et al., 2002). Moreover, SCFAs could reduce intestinal inflammation, which improves the absorptive capacity of intestine, and increases porcine feed efficiency. Interestingly, *L. casei* was also identified to enrich in the high feed-efficiency pigs. As a probiotics, *Lactobacillus* can promote intestinal development and metabolism. The study in chicken showed that *Lactobacillus johnsonii BS15* promotes growth performance and lowers fat deposition (Wang et al., 2017).

*M. versatilis, P. fuorescens*, and *P. mendocina* were also enriched in the high feed-efficiency pigs in the metagenomic sequencing analysis (Supplementary Table 5, Figure 3). These bacteria have the ability to utilize amine and nitrate (Feng et al., 2012; Kalyuzhnaya et al., 2012). The KEGG pathways of glycine, serine and threonine metabolism, and valine, leucine and isoleucine degradation had the higher abundances in the low RFI pigs (Figure 4B). McCormack et al. indicated that the higher abundance of valine, leucine, and isoleucine biosynthesis pathway in the low RFI pigs may be linked to the concentration of isobutyric acid (McCormack et al., 2017). Isobutyric acid is an end-product of microbial deamination of valine and generally considered as an indicator of better utilization of dietary protein (Walsh et al., 2013). The KOs related to amino acid metabolism (K13498 and K02510) and nitrogen metabolism (K00376) were also enriched in the low RFI pigs (Figure 4A). These results suggested that the gut microbiome of high feed efficiency pigs might be more available to utilize the diet protein than that of pigs with low feed efficiency.

We observed that KEGG orthologies related to the metabolism and transport of monosaccharides were negatively associated with porcine feed efficiency (Figure 4). A previous report suggested that an obesity-associated gut microbiome had increased capacity for energy harvest (Turnbaugh et al., 2006). As we have well known, fat deposition reduces the feed efficiency in pigs (Martinsen et al., 2015). In addition, some monosaccharides such as glucose and mannose may influence polysaccharide digestion by repressing the expression of the genes encoding cellulases, chitinases, and xylanases in bacteria (Rodríguez et al., 2005). KOs related to flagellar assembly (K02393) and two-component system (K07719) were also enriched in the pigs with the low feed efficiency. These two KEGG pathways were also enriched in the distal colon microbiome of obesity mouse and human (Turnbaugh et al., 2006; Duca et al., 2014). In our previous study, we also detected that these two pathways were significantly associated with porcine fatness (Yang et al., 2016). Interestingly, the pigs clustered into the *Prevotella*-enterotype tended to have the low feed efficiency. We also identified four RFI-associated OTUs annotated to *Prevotella*, including *P. copri*. The study in humans demonstrated that *P. copri* could induce insulin resistance (Pedersen et al., 2016). Taken together, the gut microbiome of pigs with low feed efficiency may contribute to the development of host fatness that reduces feed efficiency.

In conclusion, we detected 31 OTUs that were potentially linked to porcine feed efficiency by an OTU-based association analysis in the 280 Duroc pigs. Our results suggested that the gut microbiome of pigs with the high feed efficiency have a greater ability to utilize the dietary polysaccharides and protein. We inferred that gut microbiota might improve porcine feed efficiency through promoting intestinal health by the SCFAs produced by fermenting dietary polysaccharides. However, the functional capacities of gut microbiome inducing fatness might reduce porcine feed efficiency. These results provide important insights into how gut microbiome influences porcine feed efficiency, and gave the basic knowledge for improving porcine feed efficiency through modulating the gut microbiota in pig industry.

## Author contributions

LH: conceived and designed the experiments, revised the manuscript; CC: conceived and designed the experiments, analyzed the data, wrote and revised the manuscript; HY: performed the experiments, analyzed the data and wrote the manuscript; ZZ: analyzed the data; ZW and MY: measurement of pig phenotypes; XH, SF, MH, and YZ: collected the samples. All authors read and approved the final manuscript.

### Conflict of interest statement

The authors declare that the research was conducted in the absence of any commercial or financial relationships that could be construed as a potential conflict of interest.

## Supplementary material

Supplementary Figure 1 Phenotypic distribution of the 18 samples used in metagenomic sequencing.

Supplementary Figure 2 Taxonomic distribution of bacterial phyla and genera obtained from 16S rRNA gene sequencing.

Supplementary Table 1 Phenotypic characteristics of the experimental pigs used in this study.

Supplementary Table 2 Summary of metagenomic sequencing data.

Supplementary Table 3 The identified bacterial species not belonging to the host-associated organisms.

Supplementary Table 4 OTUs showing the tendency associated with porcine RFI (*P* < 0.01, FDR < 0.1).

Supplementary Table 5 Bacterial species showing the tendency associated with porcine RFI with metagenomic sequencing data (FDR < 0.2).
