# Supplementary figures and images for: Unraveling the Fecal Microbiota and Metagenomic Functional Capacity Associated with Feed Efficiency in Pigs
Source: Front Microbiol. 2017 Aug 15;8:1555. doi: 10.3389/fmicb.2017.01555 (PMC5559535; doi:10.3389/fmicb.2017.01555)

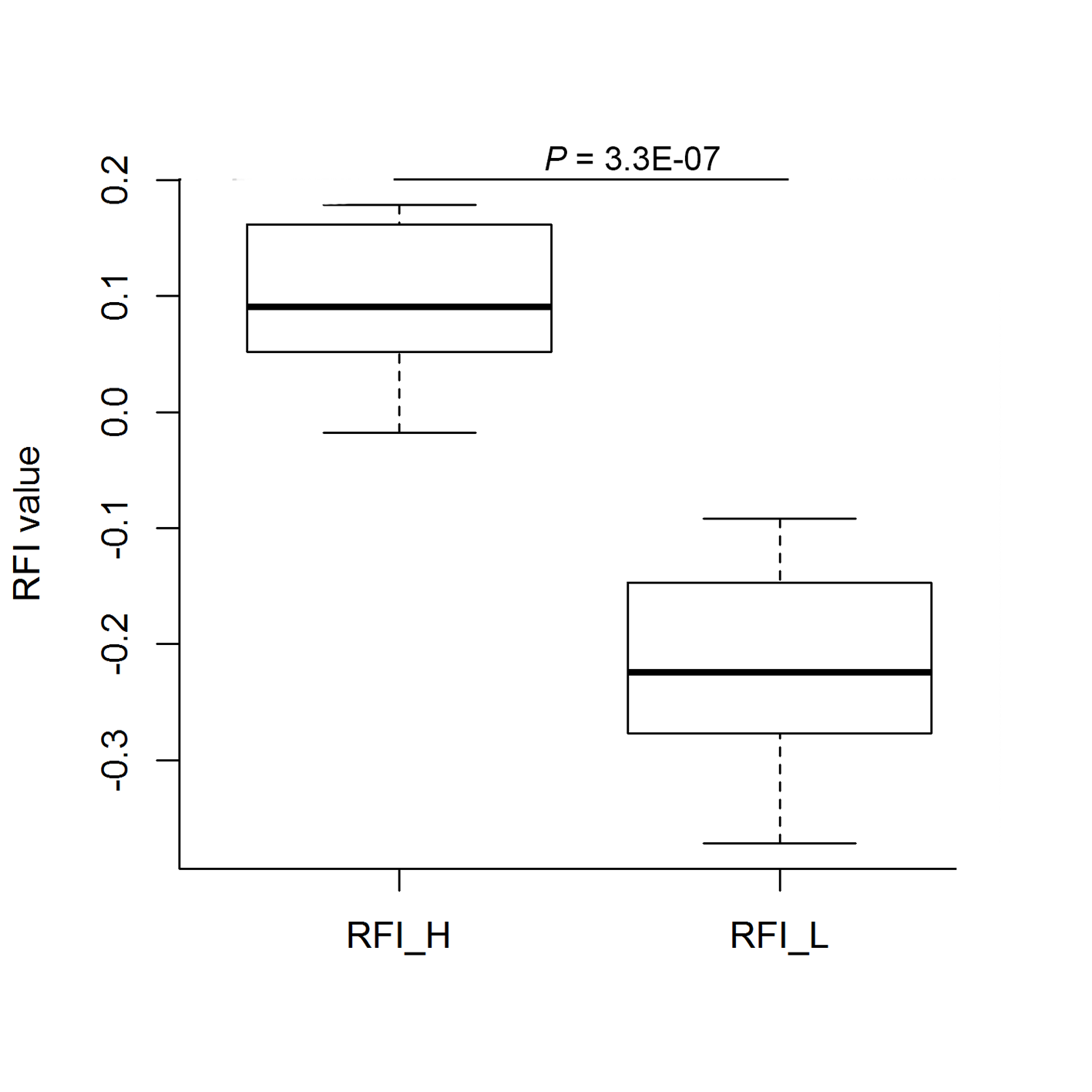

Supplement: Supplementary Figure 1 — Phenotypic distribution of the 18 samples used in metagenomic sequencing. [file Image1.TIFF]

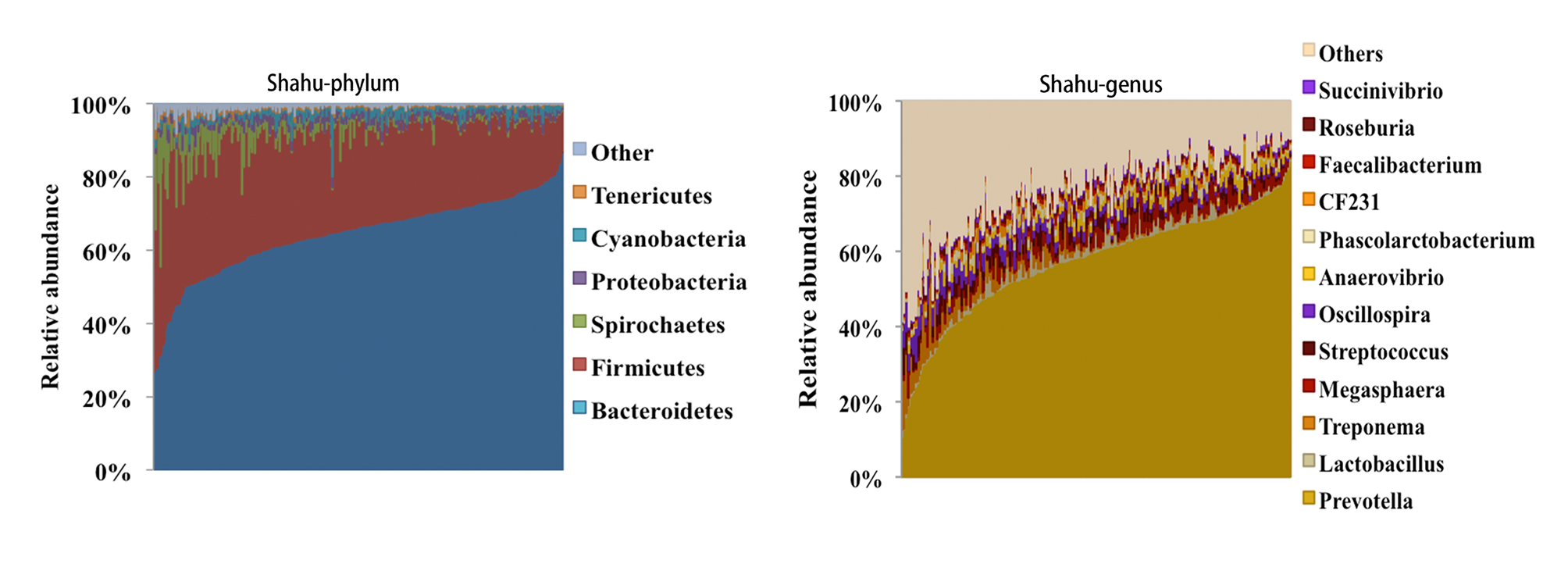

Supplement: Supplementary Figure 2 — Taxonomic distribution of bacterial phyla and genera obtained from 16S rRNA gene sequencing. [file Image2.TIF]
